# Are Pharmaceutical Company Payments Incentivising Malpractice in Japanese Physicians?

**DOI:** 10.15171/ijhpm.2019.60

**Published:** 2019-07-24

**Authors:** Yurie Kobashi, Makoto Watanabe, Hideaki Kimura, Asaka Higuchi, Akihiko Ozaki

**Affiliations:** ^1^Medical Governance Research Institute, Tokyo, Japan.; ^2^Department of Anesthesia, Jyoban Hospital of Tokiwa Foundation, Iwaki, Japan.; ^3^Waseda Chronicle, Tokyo, Japan.; ^4^Department of Breast Surgery, Jyoban Hospital of Tokiwa Foundation, Iwaki, Japan.

## Dear Editor,

Pharmaceutical industry (Pharma) funding plays a pivotal role in medical progress but has resulted in the extensive commercialization of clinical and academic research.^1^ Profit-driven interest from Pharma has been demonstrated to have a negative impact on the integrity and objectivity of science, research publications and patient management.^2,3^ Pharma is generally held in very low esteem, especially in wealthier nations, with widespread claims of secrecy and bribery and corruption in a relentless pursuit of profits. There is a perceived non-disclosure of data, particularly from clinical trials, plus the marketing focus of Pharma companies is seen as compromising medical education and improperly influencing prescription practices. Pharma spends on about twice as much on marketing as on research and development of new drugs. Furthermore, much ‘research’ is disguised as science whereas it is actually for marketing purposes. Scientists, physicians and regulators are tainted by being recognized as complicit in these activities.

In 2004, the World Medical Association proclaimed that conflicts of interest between Pharma and physicians can adversely affect patient care as well as the reputation of the medical profession. Physicians are supposed to objectively decide what is best for the patient, while Pharma aims for financial profit for their shareholders by selling their own products and out-competing rivals. Commercial incentives can thus jeopardise a physician’s objectivity. However, instead of prohibiting relationships between physicians and Pharma, the World Medical Association advises establishing self-policing guidelines to govern such relationships. These must encompass the key principles of disclosure, avoidance of obvious conflicts of interest, and safeguarding the physician’s autonomy to act in the best interests of their patients. Physician/Pharma interactions and acceptance of gifts from a company are known to affect physicians’ prescribing behavior and contribute to irrational prescribing of the company’s products.^4^ Thus, it is imperative to encourage complete transparency in Pharma/physician relationships. The Physician Payments Sunshine Act, enacted in the United States in 2010, marked a first step toward accomplishing this. And since 2014, the government’s Center for Medicare and Medicaid Services has been reporting payment information to healthcare providers and educational hospitals on the Open Payment website (https://www.cms.gov/openpayments/), to which the general public has unlimited access. Furthermore, the American Medical Association has repeatedly and extensively stressed the necessity for transparency in financial relationships between Pharma companies and physicians.^5^

In Japan, steps are also being taken to rectify the problem. The Japan Pharmaceutical Manufacturers Associations (JPMA) created the ‘Transparency Guideline for the Relation between Corporate Activities and Medical Institutions’ in 2011. This requires the 78 pharmaceutical companies belonging to the JPMA to disclose details of all their payments to healthcare providers, together with their names and affiliations. Each company should itemize their payment details on its own website, in a timely fashion, with open access. Unfortunately, formats for the data have not been specified, there is no uniformity, and so much is unclear and not easily comparable. Thus, it has not been possible for researchers, journalists or the public to obtain or analyze data easily. The JPMA’s latest 2018 guideline update has not improved things in this respect. This is surprising given that the JPMA experienced infamous scientific misconduct with the Valsartan Scandal, in which an employee of Novartis was deeply involved in data fabrication in multiple clinical trials, leading to the retraction of all main academic articles on the drug.^6^ Similarly, healthcare providers have not actively sought to change their relationships with Pharma, possibly because a majority of high-ranking physicians, such as medical school professors, have been benefitting substantial from the cozy financial relationships.

To provide increased transparency, we have created a uniform database of Pharma payments to physicians in Japan, using the payment data disclosed on 78 Pharma company websites. A comprehensive analysis of the payment data with respect to Executive Board members of Professional Medical Associations,^7^ Clinical Practice Guidelines authors,^8^ and researchers involved in the Valsartan Scandal,^6^ revealed their significant financial support from Pharma. The database has been available to all on the Money Database website (http://db.wasedachronicle.org/) since January 15, 2019. So far, it has attracted more than 2 500 000 page views, engendering significant media coverage. While it is too soon to know whether or not the initiative has enhanced the health and well-being of patients, or whether it is possible to measure this, the new database has certainly helped raise awareness of this controversial issue among the general population.

It is clear that Pharma has a herculean task to rebuild trust, in Japan, the United States and in many other parts of the world, mostly in the wealthier nations. An extensive global survey in 2018 found that only 55% of people trusted Pharma, a figure which had remained constant for a decade [Edelman Trust Barometer 2018. https://www.edelman.com/sites/g/files/aatuss191/files/2018-10/Edelman_Trust_Barometer_Global_Healthcare_2018.pdf]. In the United States, unacceptably high drug prices, the opioid scandal, class-action cases against perceived Pharma wrongdoings, and other factors are actually threatening the actual existence of several Pharma companies. Despite the 2010 Physician Payments Sunshine Act and other initiatives, US public trust in Pharma fell from 51% in 2017 to 38% in 2018, condemning the industry to the “distrusted” category. In Japan, trust in Pharma among the general public had risen 14 points to 68% in the same timeframe.

Despite the obvious potential ethical violations in Pharma/physician linkages, it is unlikely that the new Japanese database will have much impact on changing them, as the JPMA guidelines are not mandatory or enforced, and there are no penalties involved for non-compliance. The JPMA’s position is “we hope that the medical institutions and medical professionals will kindly understand the purpose of this Guideline and provide their cooperation.” Moreover, Pharma companies outside the JPMA are not involved in the data sharing. There is a need to enact rigorous legislation to prevent Pharma from using their power and funding to influence the activities of physicians for their own benefits.

## Acknowledgments

We offer special thanks to Dr. Andy Crump for his constructive opinions on this work.

## Ethical issues

Not applicable.

## Competing interests

AO receive personal fees from Medical Network Systems (MNES Inc.). All other authors declare no conflict of interests.

## Authors’ contributions

YK and AO contributed to writing the paper. AO, MW, HK, and AH contributed to study design and data collection.

## Authors’ affiliations

^1^Medical Governance Research Institute, Tokyo, Japan.^2^Department of Anesthesia, Jyoban Hospital of Tokiwa Foundation, Iwaki, Japan.^3^Waseda Chronicle, Tokyo, Japan.^4^Department of Breast Surgery, Jyoban Hospital of Tokiwa Foundation, Iwaki, Japan.
